# Moral distress in healthcare professionals working with motor neuron disease

**DOI:** 10.1017/S1478951526103058

**Published:** 2026-07-15

**Authors:** Megan Walls, Austin Claffey, Miriam Galvin

**Affiliations:** 1 Academic Unit of Neurology, Trinity College Dublin, Dublin, Ireland; 2 Institute of Health and Social Care, London South Bank University, London, UK

**Keywords:** Moral distress, professional quality of life, motor neuron disease, amyotrophic lateral sclerosis, workforce

## Abstract

**Objectives:**

To (1) identify clinical situations that may contribute to the experience of moral distress (MD) among professionals working with motor neuron disease (MND), (2) measure the occurrence and intensity of MD, and (3) explore associations with professional quality of life, turnover intention, and associated risk and/or protective factors.

**Methods:**

A cross-sectional online survey was distributed to healthcare professionals working in MND services across Europe. Data were analyzed using descriptive and inferential statistics.

**Results:**

In total, 230 responses from professionals across 17 European countries were analyzed from the international survey. And 67% of respondents indicated that MD resonated with their experience of working with MND. Those who considered leaving or changing their position due to the challenges associated with caring for this patient population were also more likely to report resonance with MD (χ^2^ = 7.772, *p* = 0.020). The intensity of MD was associated with reduced professional quality of life (burnout [β = 0.106, *p* < 0.05], and secondary traumatic stress [β = 2.881, *p* < 0.001]). A total of 24 clinical scenarios were identified as potential contributors to experiences of MD in this population. Across all professional groups, service-/organization-level factors were the most common and distressing barriers to providing effective MND care.

**Significance of results:**

This study demonstrates that MD is experienced by healthcare professionals working with MND across Europe. MD was associated with reduced professional quality of life and increased intentions to leave or change positions, underscoring its potential implications for workforce retention and sustainability. The findings show that system/organization, patient/condition and family-level causes are the primary drivers of MD in this population. Future research should focus on evaluating the effectiveness of interventions designed to address these key drivers and mitigate the impact of MD among healthcare professionals working with MND.

## Introduction

Moral distress (MD) is recognized as a global issue affecting healthcare professionals, with significant negative consequences for both individuals and organizations (Tigard 2018). First introduced by Jameton (1984) as the experience of “*knowing the right thing to do, but being constrained from acting on it,*” the concept of MD has evolved considerably through ongoing theoretical and empirical development. Morley et al. (2019) identified more than 20 definitions, concluding that MD consistently involves 3 conditions: (1) a moral event, (2) psychological distress, and (3) a direct causal link between the two. Unlike stress, burnout, or compassion fatigue, MD specifically threatens moral integrity and arises in ethically complex situations (Morley et al. 2019; Dean et al. 2020).

Although MD is conceptually distinct from other indicators of professional well-being, it has garnered increasing international attention due to its consistent association with negative psychological and occupational outcomes among healthcare professionals. Persistent experiences of MD are associated with burnout, including emotional exhaustion and reduced professional efficacy (Whitehead et al. 2015; Rushton 2017; Xue et al. 2024). MD is also associated with heightened secondary traumatic stress (STS), as professionals may internalize the suffering of patients and families, they feel powerless to help (Lamiani et al. 2017). Moreover, high levels of MD have been identified as a strong predictor of turnover intention (Austin et al. 2017; Epstein et al. 2019; Kim et al. 2023). Therefore, MD poses a threat not only to the well-being of individual professionals but also to the sustainability of the motor neuron disease (MND) healthcare workforce.

Although initially described among critical care nurses (Corley 1995), evidence now demonstrates that MD is prevalent across many disciplines (Houston et al. 2013; Whitehead et al. 2015), particularly in contexts involving prolonged patient suffering (Rushton et al. 2013), complex end-of-life decision-making (St Ledger et al. 2013; Mehlis et al. 2018), and high-intensity clinical environments (Wall et al. 2016). Epstein et al. (2019) suggest that “*root causes*” of MD in healthcare professionals can occur at 3 levels, outlining patient-level causes (e.g., pressure to continue aggressive treatment with limited benefit), team-level causes (e.g., staffing shortages that compromise the ability to deliver safe or compassionate care), and system-level causes (e.g., institutional policies that limit access to essential resources or services). Although valuable, this framework primarily emphasizes external drivers, potentially underestimating internal or professional-level causes. Scholars such as Rushton (2017), Fourie (2015) and Pauly et al. (2012) emphasize that internal contributors, including heightened moral sensitivity, limited knowledge or confidence, self-doubt, self-blame, and individual coping styles, can shape how professionals experience ethically challenging situations, amplifying or moderating their experience of MD, particularly in complex and emotionally demanding care contexts.

Healthcare professionals working with MND may be at heightened risk of MD due to the progressive and incurable nature of the disease, which often involves witnessing continuous patient deterioration despite their best efforts to provide supportive care (Connolly et al. 2015). The time-sensitive need for aids, interventions, and support is frequently undermined by delays, resource limitations, or bureaucratic barriers, leaving professionals unable to deliver care in line with their professional values (Walls et al. 2024). In addition, reliance on often highly burdened informal caregivers, complex decision-making about supportive interventions and end-of-life care, and frequent exposure to profound patient and family suffering all contribute to emotionally and ethically challenging situations that increase the likelihood of MD for those working with this population (Artioli et al. 2025).

A recent scoping review found that clinicians working with MND frequently encounter moral and ethical dilemmas in decision-making, care planning, communication, and care delivery (Walls et al. 2024). The review identified multiple barriers that limit clinicians’ ability to provide adequate or desired care, such as patient or condition factors, family dynamics, challenges in multidisciplinary collaboration, and organizational constraints, alongside significant emotional tolls (Walls et al. 2024). This aligns with research on MD in palliative care (Maffoni et al. 2019), suggesting that those caring for individuals and families affected by MND are particularly vulnerable. Evidence from other clinical contexts further indicates that factors contributing to MD can vary across disciplines (Whitehead et al. 2015; Tomi Omoya et al. 2024) and care settings (Giannetta et al. 2021; Guariglia et al. 2023), underscoring its dynamic and context-dependent nature and the importance of adopting an exploratory research approach to capture the diverse factors that may contribute to MD in this population.

MD and its causes and consequences remain underexplored in the context of progressive neurological conditions such as MND and this gap is particularly notable given that providing care for these patients is widely recognized as ethically complex, emotionally demanding, and often characterized by uncertainty, patient deterioration, and challenging decision-making (Connolly et al. 2015; Lerum et al. 2017; Cox et al. 2025). The lack of research on this topic presents a significant barrier to understanding and addressing the distinctive challenges faced by professionals providing care for those living with this condition. Addressing this research gap is important for identifying contributing factors, developing targeted interventions, safeguarding professional well-being, and supporting long-term workforce sustainability (Currie and Laing 2023).

Therefore, the aim of this study was to (1) estimate the occurrence and intensity of MD; (2) identify the most frequent and distressing contributing factors; and (3) examine associated risk and protective factors as well as the impact of MD on professional quality of life and workforce.

## Method

### Online survey

A cross-sectional survey was developed to collect demographic and work-related data and to measure MD and professional quality of life among healthcare professionals working in MND services across Europe. The survey was informed by findings from a preceding qualitative phase. Expert input was also sought from healthcare professionals in specialist MND roles, and the survey was pilot tested with 5 professionals from the UK, Ireland, and Denmark before distribution.

#### Demographic and work-related data

Respondents provided demographic and work-related data, including employment status (full-time or part-time), the proportion of their working week devoted to MND care, professional role, gender, age, country of employment, work setting (inpatient, outpatient, or community), and years of experience in MND care. Additional items explored workforce factors such as current and past intentions to leave or change position, MND-related absenteeism, and future career plans.

#### MD assessment measures

##### MD and clinical scenarios

Respondents were first asked whether they had previously heard of the concept MD in a dichotomous yes/no question. They were then presented with a brief description of the concept and asked whether it resonated with their experience of working with MND.

Next, respondents were presented with 24 statements developed from a preceding qualitative phase (Table 1) (The findings from this qualitative study will be published elsewhere). These 24 statements were generated based on clinical situations described by professionals working with MND as potentially contributing to MD and were categorized at the professional, patient/condition, family, team, and system levels. Survey respondents were asked to indicate whether they had encountered each statement in their work (yes/no). For those marked “yes,” participants were further asked to select the 5 statements they found most distressing. An optional open-text box was included for additional comments.

**Table 1.** Potential causes of moral distress for healthcare professionals working with MND Table 1 long description.

Number	Statement
1.	Feel unable to effectively plan for the future care of individuals with ALS/MND because of my uncertainty regarding the trajectory of disease progression.
2.	Feel pressure to provide care/intervention that I don’t believe will benefit the individual with ALS/MND at the request of others, such as family, senior colleagues, or other services.
3.	Feel unprepared to engage in future care planning or end-of-life care conversations due to a lack of appropriate training.
4.	Feel that by initiating future care planning, I am adding to the individual’s psychological distress.
5.	Feel unable to offer individuals with ALS/MND and their families the emotional support or comfort they need.
6.	Have difficulty communicating hope or optimism for individuals with ALS/MND with no available cure for the disease.
7.	Worry that the wishes of individuals with ALS/MND are not being respected when problems with their communication arise.
8.	Feel unable to provide an intervention that could benefit the individual with ALS/MND because of cognitive or behavioural changes.
9.	Witness physical care needs that remain unaddressed because of difficulties with psychological adjustment.
10.	Witness individuals with ALS/MND in distress or pain due to poorly managed symptoms.
11.	Struggle to provide the necessary care for someone whose condition is already at an advanced stage when I first meet them.
12.	Feel unable to effectively deliver care for an individual with ALS/MND due to a breakdown in the patient–clinician relationship.
13.	Feel unable to alleviate the burden on family and informal caregivers because of insufficient time or available services.
14.	Struggle to meet the needs of both individuals with ALS/MND and their family members because of their conflicting priorities or opinions.
15.	Have difficulty implementing care or interventions without family or informal caregiver support.
16.	Witness compromised care for individuals with ALS/MND because of poor communication between healthcare professionals.
17.	Witness care for individuals with ALS/MND being negatively affected because of staffing vacancies within a team or service.
18.	Have difficulty providing an intervention due to reliance on another healthcare professional/organization also involved in the person’s care.
19.	Struggle to meet the needs/expectations of individuals with ALS/MND or their families due to limitations imposed by my organization’s policies or procedures.
20.	Witness individuals with ALS/MND struggling while waiting for necessary aids, devices, or services.
21.	Experience individuals with ALS/MND receiving unequal access to services/resources depending on the region where they live.
22.	Witness compromised care for individuals with ALS/MND due to lack of equipment/resources/bed capacity.
23.	Feel restricted to prioritize only certain aspects of care for individuals with ALS/MND due to limited time during appointments.
24.	Be required to care for more individuals with ALS/MND than I can safely care for.

ALS, amyotrophic lateral sclerosis.

The 24 statements were generated from analysis of the focus group data and identified as potential causes of MD for professionals working with MND. These statements were included in the online survey to assess the most frequently encountered and distressing barriers to delivering optimal care for people and families living with MND. Statements 1–6 address potential causes of MD at the professional level; statements 7–12 at the patient or condition level; statements 13–15 at the family level; statements 16–18 at the team level; and statements 19–24 at the system or organizational level.

##### MD Thermometer

The MD Thermometer was then used to assess the “real-time” intensity of MD experienced by professionals working in MND care ‘Permission was obtained from the author Dr Lucia Wocial, to use the MD Thermometer in this study’ (Wocial and Weaver 2013). The MD Thermometer is a single item, 11-point visual analogue scale, with scores ranging from 0 to 10. There are 6 descriptors provided at every second interval, ranging from 0 = “None” to 10 = “Worst Possible,” which support respondents to anchor the intensity of MD experienced. The MD Thermometer provides a standard definition of MD and respondents are asked to reflect on their clinical practice over the past 2 weeks and indicate, on the visual analogue scale, the level of MD they had experienced in their work with the MND population.

#### Professional quality of life scale – version 5 (ProQOL-5)

The Professional Quality of Life Scale-version 5 (ProQOL-5) is a tool designed to assess the impact of caregiving on professional quality of life. It is used to measure of both the positive and negative effects of working in helping professions and with people who have experienced extremely stressful events ‘Permission was obtained from the ProQOL office to use the ProQOL version 5 in this study.’ (Stamm 2010). It measures compassion satisfaction, which reflects the positive aspects of caregiving, and compassion fatigue which measures the negative impact of caregiving on professionals, and is reflected by 2 factors, burnout and STS. Each factor is assessed through 10 individual items. Stamm (2010) reported good internal consistency for the ProQOL-5 subscales, with Cronbach’s alpha values of 0.88 for compassion satisfaction, 0.75 for burnout, and 0.81 for STS.

### Online survey distribution

The survey was hosted on Qualtrics^xm^ online survey platform and was distributed to relevant gatekeepers from the Motor Neurone Disease Association (MNDA, UK) MND Professionals Community of Practice, TRICALS European Research Network and the International Alliance for MND – European Members/EUpALS, via an anonymous web link between November 2024 and January 2025. These organizations were chosen for their direct access to professionals working within specialist MND services. Gatekeepers distributed the study invitation, participant information leaflet and survey link to relevant healthcare professionals on their mailing lists. All responses were anonymous, and informed consent was obtained electronically at the beginning of the survey.

### Survey data analysis

Data were analyzed in R version 4.4.3 (R Core Team 2025), using both descriptive and inferential statistics.

#### Missing data

Missingness maps were generated to visualize the extent and pattern of missing data, and indicated that 2.2% of data were missing overall, representing a very small or “*negligible*” percentage (Mirzaei et al. 2022). Survey drop-off was random and not associated with any demographic or work-related variable or survey question. The data were treated as missing completely at random and pairwise deletion or available case analysis was used during statistical analysis (Mirzaei et al. 2022).

#### Distribution and normality

The Shapiro–Wilk test indicated that data for the primary outcome measure, the MD Thermometer Scale, were not normally distributed (*p* < 0.0001). Histograms were also used to visually assess the distribution of all variables.

#### Statistical analysis

Descriptive statistics were calculated for demographic, work-related, and outcome measure variables. Continuous variables were summarized using means (M) and standard deviations (SD) for normally distributed data, and medians (Mdn) with interquartile ranges (IQR) for non-normally distributed data. Chi-squared tests of independence were performed to assess the relationships between all categorical demographic and work-related variables, where the expected cell frequency was sufficiently large (at least 5 in each cell). Spearman’s rank-order correlation was used to assess the strength and direction of relationships between continuous variables and Kruskal–Wallis and Mann–Whitney *U* tests were used for bivariate analysis to examine differences in MD intensity scores across groups on the MD Thermometer Scale. Dunns’ post hoc test with a Bonferroni correction was used to determine significant pairwise comparisons for any significant Kruskal–Wallis’ test results. Variables demonstrating significant or near-significant associations were considered for inclusion in the multivariable model. Multiple linear regression analysis determined the independent effects of multiple predictors on Moral Distress Intensity, while controlling for potential confounding variables. Log transformations were applied as necessary to meet the assumption of linearity. Assumptions of linearity, multicollinearity, and homoscedasticity were assessed, with no violations detected in the presented model. To account for multiple comparisons, Bonferroni-adjusted *p*-values and confidence intervals are reported, with significance determined at an alpha level of 0.05.

## Results

There were 275 survey responses, “completeness” was defined as having >75% of questions answered. Thus, 230 complete surveys from healthcare professionals currently working in an MND service in Europe were included in the analysis.

### Respondents

The majority of respondents were female (*n* = 175, 76%), working full-time (*n* = 174, 76%), in the 31–50 age category (*n* = 145, 63%), and had up to 10 years of experience working with MND (*n* = 133, 58%). On average, 49.5% of their working week was with MND patients. Respondents came from 17 European countries and from 11 professional backgrounds (Table 2).

**Table 2.** The professions and countries represented (*n* = 230) Table 2 long description.

Variable	N (%)
**Profession**	
Medical	70 (30.5)
Nursing	46 (20)
Nurses	41 (18)
Nursing Assistants	5 (2)
Allied Healthcare Professionals	114 (49.5)
Physiotherapists	39 (17)
Occupational Therapists	23 (10)
Speech and Language Therapists	22 (9.5)
Psychologists	14 (6)
Dietitians	4 (2)
Social Workers	4 (2)
MND Care Co-ordinators	6 (2.5)
Psychomotor Therapists	2 (1)
**Country**	
United Kingdom	63 (27)
France	54 (23.5)
Republic of Ireland	22 (9.5)
Denmark	24 (10.5)
Sweden	11 (5)
Netherlands	8 (3.5)
Italy	13 (5.5)
Belgium	4 (1.7)
Czechia	1 (0.4)
Germany	4 (1.7)
Iceland	9 (4)
Norway	1 (0.4)
Poland	4 (1.7)
Slovenia	3 (1.3)
Spain	5 (2)
Switzerland	1 (0.4)
Türkiye	3 (1.3)

Note: Professional subgroups used for statistical analysis - Respondents’ professions were categorized into Medical (n = 70), Nursing (n = 46) and Allied Healthcare Professional (AHP) (n = 114) groups, for the purpose of statistical analysis

A total of 60 respondents (26% of complete survey responses) had considered or were currently considering leaving or changing their position because of factors related to working with MND.

### Demographic and work-related differences between professional groups

Respondents’ professions were categorized into Medical (*n* = 70), Nursing (*n* = 46) and Allied Healthcare Professional (AHP) (*n* = 114) groups, for the purpose of statistical analysis (Table 2).

Those categorized as medical were more likely to be male (Χ^2^ = 16.672, *p* < 0.001), working full-time (Χ^2^ = 8.259, *p* < 0.05), in the 51+ age category (Χ^2^ = 20.136, *p* < 0.001), and have 21+ years of experience (Χ^2^ = 35.535, *p* < 0.001), compared to nurses or AHPs.

Among nursing, a higher percentage of their working week was spent with MND patients (70%) compared to, AHPs (30%) or medics (40%) (Χ^2^ = 19.906, *p* < 0.001). AHPs were more likely to be in the under 30 age category (Χ^2^ = 20.136, *p* < 0.001), the 0–10 years of experience category (Χ^2^ = 35.535, *p* < 0.001) and work part-time (Χ^2^ = 8.259, *p* < 0.05), compared to medics and nurses. No other statistically significant differences were found in demographic or work-related variables across professional groups (Table 3).

**Table 3.** Demographic and work-related data Table 3 long description.

Variable	Overall	Medical	Nursing	AHP’s	*p*-value
	(*n* = 230)	(*n* = 70)	(*n* = 46)	(*n* = 114)	
	Mdn (IQR)	Mdn (IQR)	Mdn (IQR)	Mdn (IQR)	
Percentage of working week with individuals living with MND	41 (60)	41 (54)	**70 (50)**	31(40)	< 0.001
	*N* (%)	*N* (%)	*N* (%)	*N* (%)	
**Working Pattern**					
Full-time	174 (75.7)	**61 (87)**	35 (76)	**78 (68.5)**	<0.05
Part-time	56 (24.3)	**9 (13)**	11 (24)	**36 (31.5)**	
**Gender**					
Male	53 (23)	**28 (40)**	7 (15)	18 (16)	<0.001
Female	175 (76)	**41 (58.5**)	39 (85)	95 (83)	
Nonbinary/Third gender	2 (1)	1 (1.5)	0	1 (1)	
**Age Category**					
Under 30	21 (9)	**0 (0)**	4 (8.5)	**17 (15)**	<0.001
31−50	145 (63)	43 (61.5)	25 (54.5)	77 (67.5)	
51+	64(28)	**27 (38.5)**	17 (37)	**20 (17.5)**	
**Years of Experience**					
0−10	133 (58)	**21(30)**	31(67)	**81(71)**	<0.001
11−20	59 (26)	26 (37)	9 (19.5)	24 (21)	
21+	38 (16)	**23 (33)**	6 (13)	**9 (8)**	
**Currently Considering Leaving**	(n = 228)				
Yes	16 (7)	3 (4)	2 (4)	11 (10)	–
No	212 (93)	67(96)	44 (96)	101 (90)	
**Considered Leaving in the Past**	(*n* = 212)				
Yes	42 (20)	12 (18)	10 (23)	20 (20)	0.824
No	170 (80)	55 (82)	34 (77)	81 (80)	
**Period of Absenteeism**	(*n* = 226)				
Yes	28 (12.5)	7 (10)	7 (16)	14 (12.5)	0.646
No	198 (87.5)	63 (90)	37 (84)	98 (87.5)	
**Intention to Work with MND in the Future**	(*n* = 226)				
Yes	213 (94)	70 (100)	40 (91)	103 (92)	–
No	13 (6)	0 (0)	4 (9)	9 (8)	
**Heard of Moral Distress**	(*n* = 227)				
Yes	107 (47)	35 (50)	23 (52)	49 (43)	0.511
No	120 (53)	35 (50)	21 (48)	64 (56.6)	
**Resonate with Moral Distress**	(*n* = 226)				
Yes	152 (67)	39 (55)	30 (70)	83 (75.5)	0.101
No	46 (20.5)	19 (27)	10 (23)	17 (15)	
Not sure	28 (12.5)	12 (17)	3 (7)	13 (11.5)	

*p* values from Kruskal–Wallis and Chi-square tests are presented to show differences between professional groups on each demographic and work-related variable. Bold text highlights categories with significantly higher or lower observed counts than expected. Mdn = median, IQR = interquartile range.

### Moral distress

#### The occurrence of MD in professionals working with MND

**Figure 1.**
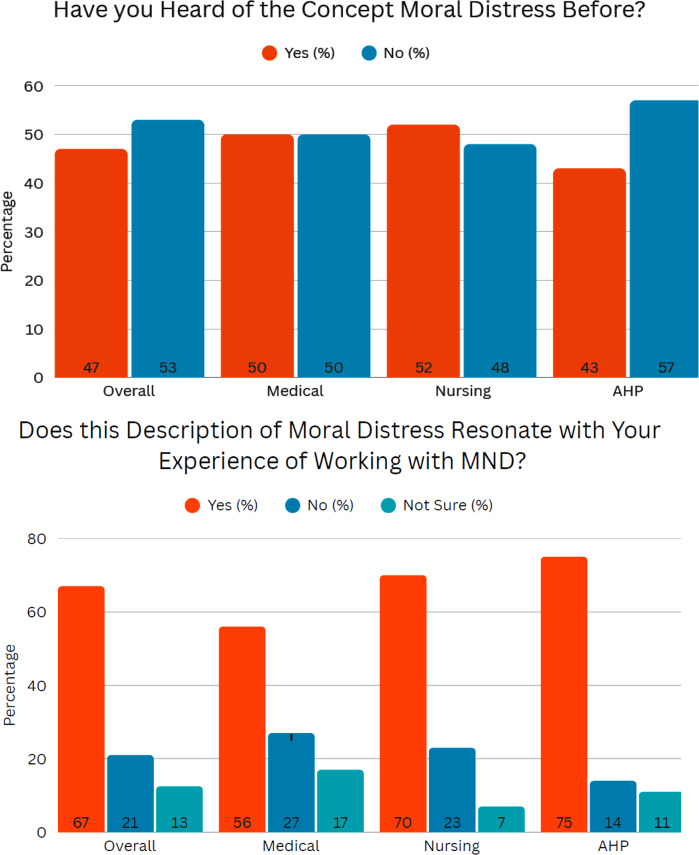
Bar charts representing awareness and resonance with moral distress in MND professionals. Figure 1 long description.

In this study, 47% (*n* = 107/227) of respondents identified having heard of the concept MD before and 67% (*n* = 152/226) indicated “*yes*,” to the description provided, that MD resonated with their experience of working with MND (Fig. 1). A higher percentage of AHPs (75%) and nurses (70%) felt that MD resonated with their experience of working with MND compared to medics (56%) (Fig. 1).

#### Intensity of MD among professionals working in MND care

**Figure 2.**
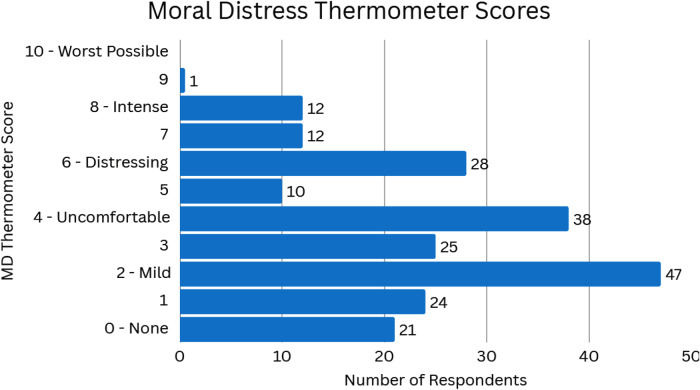
Intensity of moral distress reported by all respondents on the MD Thermometer Scale.

The overall median MD Thermometer score was 3 (IQR = 3). In terms of MD experienced over the previous 2 weeks, most respondents reported levels described as either *mild* (21%) or *uncomfortable* (18%). However, one-quarter (25%) reported levels of distress rated as *distressing* or higher, with 6% selecting *intense*. Only 10% of respondents indicated experiencing no MD in the previous 2 weeks (Fig. 2).

#### The most frequent factors potentially contributing to MD among MND professionals

Respondents were asked to select “Yes” or “No” to whether they had experienced each of 24 statements listed in Table 1 in their work with people living with MND. The median number of “Yes” responses was 14/24 (IQR = 7). The 67% of respondents who identified with the experience of MD endorsed a greater number of these statements (Mdn: 16, Χ^2^ = 33.737, *p* < 0.001) and reported higher intensities of distress on the MD Thermometer Scale (Mdn: 4, Χ^2^ = 33.722, *p* < 0.001), than those who did not resonate with MD or selected “unsure” (Table 4).

**Table 4.** Median MD thermometer score and number of scenarios endorsed, by resonance with the moral distress description (yes/no/not sure) in MND practice Table 4 long description.

Response category – Resonate with Moral Distress?	Yes	No	Not sure	Χ^2^ Test results (*p*-value)
*N* (%)	128 (67)	46 (20.5)	28, (12.5)	–
Median Number of Statements Selected “Yes”	16	10	10	*p* < 0.001
Median MD Thermometer Score	4 (Uncomfortable)	2 (Mild)	2 (Mild)	*p* < 0.001

#### Factors contributing to MD

The statement most frequently experienced as potential contributor to MD was the same across all professional groups:

1. “*Witness individuals with MND struggling while waiting for necessary aids, devices, or services”* (experienced by 89% of total respondents)

The second most frequently experienced statement was

1. *“Experience individuals with MND receiving unequal access to services/resources depending on the region where they live”* (experienced by 77% of total respondents)

Followed by:

1. “*Witness individuals with MND in distress or pain due to poorly managed symptoms”* (experienced by 71%)
2. “*Witness physical care needs that remain unaddressed because of difficulties with psychological adjustment*” (experienced by 71%)
3. “*Feel unable to alleviate the burden on family and informal caregivers because of insufficient time or available services”* (experienced by 70%)

The frequency statistics and rank order for each of the 24 statements, both overall and broken down by professional group are presented in Table 5.

**Table 5.** The number and percentage of respondents, overall and by professional group who endorsed each statement, along with the rank order of the most frequently endorsed statements for each professional group Table 5 long description.

Statement	Overall (*n* = 220)		Medical (*n* = 69)		Nursing (*n* = 42)		AHPs (*n* = 109)		
	Yes, *n* (%)	Rank	Yes, *n* (%)	Rank	Yes, *n* %	Rank	Yes, *n* %	Rank	*p*-value
1. Feel unable to effectively plan for the future care of individuals with ALS/MND because of my uncertainty regarding the trajectory of disease progression.	113 (51)	**17**	29 (42)	**20.5**	17 (40)	**16.6**	67 (61)	**8**	0.011*
2. Feel pressure to provide care/intervention that I don’t believe will benefit the individual with ALS/MND at the request of others, such as family, senior colleagues, or other services.	105 (47)	**19**	33 (47)	**18**	23 (45)	**14**	53 (48)	**19.5**	0.933
3. Feel unprepared to engage in future care planning or end-of-life care conversations due to a lack of appropriate training.	71 (32)	**22**	14 (20)	**24**	13 (30)	**22**	44 (40)	**22**	0.019*
4. Feel that by initiating future care planning, I am adding to the individual’s psychological distress.	113 (51)	**17.5**	38 (55)	**13.5**	16 (38)	**19**	59 (54)	**16**	0.159
5. Feel unable to offer individuals with ALS/MND and their families the emotional support or comfort they need.	121 (55)	**15**	35 (50)	**17**	25 (59)	**6**	61 (55)	**14.5**	0.639
6. Have difficulty communicating hope or optimism for individuals with ALS/MND with no available cure for the disease.	126 (57)	**13**	38 (55)	**13.5**	24 (57)	**8.5**	64 (58)	**11**	0.892
7. Worry that the wishes of individuals with ALS/MND are not being respected when problems with their communication arise.	133 (60)	**7**	38 (55)	**13.5**	24 (57)	**8.5**	71 (65)	**6**	0.363
8. Feel unable to provide an intervention that could benefit the individual with ALS/MND because of cognitive or behavioral changes.	129 (59)	**9.5**	42 (60)	**11**	24 (57)	**8.5**	63 (57)	**12.5**	0.899
9. Witness physical care needs that remain unaddressed because of difficulties with psychological adjustment.	155 (71)	**3.5**	47 (68)	**6.5**	29 (69)	**5**	79 (72)	**2.5**	0.804
10. Witness individuals with ALS/MND in distress or pain due to poorly managed symptoms.	155 (71)	**3.5**	58 (84)	**3**	23 (54)	**11.5**	74 (67)	**5**	0.003**
11. Struggle to provide the necessary care for someone whose condition is already at an advanced stage when I first meet them.	130 (59)	**8**	46 (66)	**8.5**	23 (54)	**11.5**	61 (55)	**14.5**	0.301
12. Feel unable to effectively deliver care for an individual with ALS/MND due to a breakdown in the patient-clinician relationship.	62 (28)	**24**	26 (37)	**22**	11 (26)	**23.5**	25 (22)	**24**	0.098
13. Feel unable to alleviate the burden on family and informal caregivers because of insufficient time or available services.	154 (70)	**5**	47 (68)	**6.5**	32 (76)	**2.5**	75 (68)	**4**	0.619
14. Struggle to meet the needs of both individuals with ALS/MND and their family members because of their conflicting priorities or opinions.	129 (59)	**9.5**	44 (63)	**10**	16 (38)	**19**	69 (63)	**7**	0.011*
15. Have difficulty implementing care or interventions without family or informal caregiver support.	123 (56)	**14**	48 (69)	**5**	17 (40)	**16.5**	58 (53)	**17**	0.008**
16. Witness compromised care for individuals with ALS/MND because of poor communication between healthcare professionals.	127 (58)	**11.5**	46 (66)	**8.5**	18 (42)	**15**	63 (57)	**12.5**	0.048*
17. Witness care for individuals with ALS/MND being negatively affected because of staffing vacancies within a team or service.	127 (58)	**11.5**	38 (55)	**13.5**	24 (57)	**8.5**	65 (59)	**10**	0.832
18. Have difficulty providing an intervention due to reliance on another healthcare professional/organization also involved in the person’s care.	116 (53)	**16**	37 (53)	**16**	22 (52)	**13**	57 (52)	**18**	0.984
19. Struggle to meet the needs/expectations of individuals with ALS/MND or their families due to limitations imposed by my organization’s policies or procedures.	99 (45)	**20**	32 (46)	**19**	14 (33)	**21**	53 (48)	**19.5**	0.230
20. Witness individuals with ALS/MND struggling while waiting for necessary aids, devices, or services.	196 (89)	**1**	65 (94)	**1**	38 (90)	**1**	93 (85)	**1**	0.171
21. Experience individuals with ALS/MND receiving unequal access to services/resources depending on the region where they live.	170 (77)	**2**	59 (85)	**2**	32 (76)	**2.5**	79 (72)	**2.5**	0.128
22. Witness compromised care for individuals with ALS/MND due to lack of equipment/resources/bed capacity.	150 (68)	**6**	53 (76)	**4**	31 (73)	**4**	66 (60)	9	0.052
23. Feel restricted to prioritize only certain aspects of care for individuals with ALS/MND due to limited time during appointments.	92 (42)	**21**	29 (42)	**20.5**	16 (38)	**19**	47 (43)	**21**	0.854
24. Be required to care for more individuals with ALS/MND than I can safely care for.	67 (30)	**23**	23 (33)	**23**	11 (26)	**23.5**	33 (30)	**23**	0.729

Chi-squared tests were used to assess differences in responses between groups, with corresponding *p*-values reported. Blue shading indicates lower-than-expected frequencies; orange indicates higher-than-expected frequencies.

* <0.05, **<0.01, ***<0.001.

Considering the multilevel categorizations (professional, patient/condition, family, team, and service/organizational), factors at the service or organizational level were, identified as the most frequently experienced barriers to providing effective MND care, making them the most likely contributors to MD in this population. These were followed by patient/condition and family-level factors and this pattern that was consistent across all professional groups.

#### Differences in potential contributing factors to MD between professional groups

The proportion of respondents who reported experiencing 6 of the 24 clinical statements differed significantly across professional groups (Table 5). There were no significant between-group differences across the remaining statements.

#### The most distressing factors potentially contributing to MD among ALS/MND healthcare professionals

Respondents who indicated “*Yes*” to experiencing any of the 24 statements were subsequently asked to identify the 5 they found most distressing when working with individuals living with MND. The top 5 statements for each professional group are presented in Table 6, with overall results provided in supplementary material 3. Notably, every statement was selected by at least 1 respondent as among their top 5, underscoring the wide and varying range of challenges faced. When viewed through the multilevel framework, the highest-rated stressors across professional groups reflected a combination of service or organizational factors, complexities associated with the patient’s condition, and family-level challenges, indicating that MD is likely arising from barriers operating at multiple levels.

**Table 6.** Five highest rated stressors by professional group Table 6 long description.

**Medical Top 5 Stressors**
• Witness individuals with ALS/MND struggling while waiting for necessary aids, devices, or services.
• Experience individuals with ALS/MND receiving unequal access to services/resources depending on the region where they live.
• Witness compromised care for individuals with ALS/MND due to lack of equipment/resources/bed capacity.
• Feel unable to alleviate the burden on family and informal caregivers because of insufficient time or available services.
• Witness individuals with ALS/MND in distress or pain due to poorly managed symptoms.
**Nursing Top 5 Stressors**
• Witness compromised care for individuals with ALS/MND due to lack of equipment/resources/bed capacity.
• Experience individuals with ALS/MND receiving unequal access to services/resources depending on the region where they live.
• Witness individuals with ALS/MND struggling while waiting for necessary aids, devices, or services.
• Feel unable to alleviate the burden on family and informal caregivers because of insufficient time or available services.
• Feel unable to offer individuals with ALS/MND and their families the emotional support or comfort they need.
**AHPs Top 5 Stressors**
• Witness individuals with ALS/MND struggling while waiting for necessary aids, devices, or services.
• Worry that the wishes of individuals with MND are not being respected when problems with their communication arise.
• Have difficulty communicating hope or optimism for individuals with ALS/MND with no available cure for the disease.
• Feel unable to provide an intervention that could benefit the individual with ALS/MND because of cognitive or behavioral changes.
• Feel unable to alleviate the burden on family and informal caregivers because of insufficient time or available services.

### Demographic, work-related and professional quality of life factors associated with MD intensity

#### Bivariate analysis

Bivariate analysis suggested that female respondents reported higher intensities of MD, on the MD Thermometer than males (*W* = 5096.5, *p* = 0.032) as did those with a current or past intention to leave or change position (*W* = 2091, *p* = 0.002) than those who had not considered leaving or changing positions. Higher intensities of MD were weakly associated with a higher percentage of the working week spent with MND patients (*r*_s_ = 0.254, *p* = 0.0001), and more strongly associated with higher levels of burnout (*r*_s_ = 0.469, *p* < 0.001) and STS (*r*_s_ = 0.525, *p* < 0.001). While lower intensities of MD were weakly associated with higher levels compassion satisfaction (*r*_s_ = −0.248, *p* < 0.001). There were no differences observed between profession groups, age or experience categories, work patterns or work settings on the MD Thermometer Scale (Table 7).

**Table 7.** MD thermometer scores: bivariate and multivariable analyses Table 7 long description.

Outcome – Moral Distress Thermometer		
Variable	Bivariate analysis^1^ test stat (X^2^, *W, r*_s_), *p*-value	Multivariable analysis^2^ β [95% CI]
**Profession Group**	Χ^2^ = 0.694, *p* = 0.707	
AHP (ref)		^∼^
Medical		0.204 [−0.760, 1.168]
Nursing		0.863 [−0.238, 1.965]
**Gender**	*W* = 5096.5, *p* = 0.032*	
Female (ref)		∼
Male		−0.317 [−1.259,0.625]
**Experience**	Χ^2^ = 1.273, *p* = 0.529	
0−10 (ref)		∼
11−20		0.126 [−0.819, 1.072]
21+		−0.394 [−1.521, 0.734]
**% of work week with ALS/MND**	*r*_s_ = 0.254, *p* = 0.0001***	0.008 [−0.005, 0.021]
**Work Setting**		
Inpatient:	*W* = 499, *p* = 0.065	
No (ref)		∼
Yes		0.689 [−0.095, 1.474]
Outpatient:	*W* = 590, *p* = 0.471	–
Community:	*W* = 518.5, *p* = 0.929	–
Turnover Intention	*W* = 209, *p* = 0.002***	0.439 [−0.504, 1.403]
Absenteeism	*W* = 208, *p* = 0.072	−0.373 [−1.574, 0.868]
Burnout	r_s_ = 0.469, *p* < 0.001***	0.107 [0.009, 0.204]*
Secondary Traumatic Stress^log^	*r*_s_ = 0.525, *p* < 0.001***	2.887 [0.899, 4.875]***
Age	Χ^2^ = 3.674, *p* = 0.159	–
Work Pattern (Part-time/Full-time)	*W* = 4286.5, *p* = 0.723	–
Compassion Satisfaction	*r*_s_ = − 0.248, *p* < 0.001***	–

1 Bivariate nonparametric analyses were conducted to examine between-group differences on the MD Thermometer Scale, with statistical significance determined at α = 0.05.

2 Multivariable linear regression was used to further assess the impact of key variables on the primary outcome (total MD Thermometer score), adjusting for the effects of all other included predictors. Bonferroni-adjusted confidence intervals and *p*-values are reported to control for multiple comparisons. Statistical significance is indicated as follows: *<0.05, **<0.01, ***<0.001.

#### Multivariable analysis

After adjusting for demographic and work-related variables, multiple linear regression analysis indicated that only burnout (β = 0.107, *p* < 0.05) and STS (β = 2.887, *p* < 0.001) remained significant, independent predictors of the intensity of MD reported on the MD thermometer. No other variables in the model reached statistical significance (Table 7). This model explained 35% of the variance in MD Thermometer scores.

#### MD and intention to leave

In this study, 100% of respondents who were considering leaving or changing their position due to factors related to working with MND indicated that the description of MD resonated with their experience of working with this population (15/15; χ^2^ = 7.77, *p* = 0.020). Moreover, 88% of those who had previously considered leaving their position for similar reasons also resonated with experiencing MD (36/41; χ^2^ = 13.19, *p* = 0.001).

## Discussion

This is the first study to address the issue of MD with healthcare professionals working with individuals and families living with MND, or to our knowledge any progressive neurological condition. This is a European study and represents healthcare professionals from 17 different countries and 11 professional backgrounds.

### MD in healthcare professionals working with MND

In this survey, over two-thirds (67%) of respondents resonated with the concept of MD in their work with MND patients. While recognition with MD was common, MD Thermometer scores indicated generally low overall intensity. A comparable pattern was reported by Mehlis et al. (2018), among oncologists and oncology nurses involved in end-of-life decision-making.

In this study, most respondents reported experiencing MD over the previous 2 weeks at *mild* (21.5%) or *uncomfortable* (17.5%) levels, indicating that MD is common but not always intense. All participants were exposed to several potentially morally distressing scenarios. Repeated exposure to morally challenging situations can, in some cases, foster adaptive coping and enhanced ethical reasoning, resulting in what Rushton (2017) describe as moral resilience – the capacity to function effectively in complex clinical settings without severe psychological strain. However, while moral resilience may mitigate distress it does not eliminate exposure to ethically challenging circumstances or moral stressors. Moreover, Epstein and Hamric’s concepts of moral residue and the crescendo effect highlight how unresolved episodes of MD can accumulate over time with repeated exposure, increasing vulnerability to future distress (Epstein and Hamric 2009). These insights suggest that even “mild” distress, if recurrent and unaddressed, can gradually erode professional well-being, underscoring the importance of early, proactive interventions to prevent cumulative harm (Epstein and Hamric 2009; Rushton 2017).

Consistent with Mehlis et al. (2018), we also observed substantial interindividual variation, with 25% of respondents reporting levels ranging from *distressing* to *intense* over the previous 2 weeks, while only 10% reported no distress. This distribution highlights a sizeable proportion who are at risk of considerable MD and likely require targeted support, to resolve episodes of distress, promote recovery, and sustain their well-being in the workplace.

### Factors contributing to MD in healthcare professionals working with MND

We did not find any significant demographic or work-related factors associated with MD intensity in multivariable models. Although bivariate analyses suggested higher MD among females and among clinicians who spent a higher proportion of their week working with MND, these associations were not retained after adjusting for confounding variables.

Situations that can give rise to MD were identified across professional, patient/condition, family, team, and system/organizational levels. Categorizing circumstances in this way facilitates targeted identification and resolution of contributing factors and has also been proposed by other authors (Epstein et al. 2019; Maffoni et al. 2019). Notably, situations at the organizational/system level were both the most frequently encountered and the most distressing for survey respondents.

Witnessing individuals with MND struggling while waiting on necessary aids, devices or services, was both the most frequently encountered (89%) and the most distressing (rated most distressing by 39%) situation identified, across all professional groups. This reflects the uniquely time-sensitive nature of MND care, in which clinicians must anticipate and rapidly respond to evolving needs while also navigating varying levels of readiness and decision-making capacity (Hogden et al. 2012; McConigley et al. 2014). Misalignment between existing service pathways and the rapid progression of the disease can limit clinicians’ ability to meet patients’ needs effectively. This finding highlights the emotional burden for professionals, witnessing potentially avoidable suffering without having the means to prevent it. As Molinaro (2025) argues, professionals cannot deliver effective care within systems that undermine their ability to do so.

Witnessing patients and families having unequal access and levels of service depending on where they lived was experienced by 77% and rated most distressing by 34%. This inequity likely reflects both disparities in generalist provider confidence in managing MND across regions (McConigley et al. 2014) and the logistical challenges of accessing specialist services, for those with advanced disease or living in rural/remote areas (Velaga et al. 2023). These findings highlight an ethical tension, commonly experienced in MND care, when geography influences clinical outcomes and professionals witness inequities in care provision while feeling powerless to rectify them. This scenario also underscores the emotional burden on clinicians, when their ethical commitment to provide equitable care is compromised. The results point to the need for systemic solutions, such as telehealth, training and capacity building and streamlined referral pathways, to mitigate the impact of these often called “postcode lotteries” on both patients and healthcare professionals.

Patient/condition and family-level barriers were the next most frequently experienced across professional groups. Among family related challenges, the most common (70%) and most distressing (30%) was witnessing caregiver burden that professionals felt unable to alleviate. This was due to service capacity constraints and the lack of accessible, dedicated family supports. These findings reinforce existing evidence that MND family caregivers face high levels of burden and unmet need but receive limited formal support (Schischlevskij et al. 2021; Knudsen and Nikolajevic-Pujic 2025). However, these findings specifically highlight the impact on professional caregivers, who often witness pronounced family/informal caregiver burden yet feel helpless in their ability to alleviate it.

At the patient and condition level, the most frequently encountered barriers were witnessing pain or distress from difficult-to-manage symptoms (70%) and unmet physical care needs resulting from challenges in psychological adjustment (71%). These scenarios expose a core ethical tension between clinicians’ commitment to alleviating suffering and pain and the challenges imposed by an incurable, progressive disease. Professionals working with these patients must also navigate a delicate balance between respecting patient autonomy and values and providing what they consider to be the most appropriate care. This can give rise to ethical conflict and uncertainty, particularly when priorities diverge between healthcare professionals, patients, and families. Ethical dilemmas are, to some extent, unavoidable in MND care due to the disease’s complex trajectory, including its rapid progression, cognitive and communication impairments, and the emotionally charged decisions it necessitates. Consequently, while system-level strategies to address MD remain crucial, there is also a need for targeted interventions that support clinicians in recognizing, regulating, and processing their emotional responses and ethical reasoning (Rushton et al. 2013).

### Differences between professional groups

Recognition of MD varied by professional group, with higher levels reported by allied health professionals (75%) and nurses (70%) compared to medics (56%). Similar patterns have been observed in previous studies, where medical professionals consistently report lower levels of MD than their nursing or allied health counterparts (Whitehead et al. 2015; Dodek et al. 2016; Neumann et al. 2018; Epstein et al. 2019). This disparity is frequently attributed to hierarchical team structures (Dodek et al. 2016), lower levels of direct patient care typically provided by medical staff (Whitehead et al. 2015; Dodek et al. 2016), and a professional culture within medicine that often values emotional detachment or suppression (Paddison 2016). However, given the limitations in treatment options for MND and the complex ethical challenges it presents, the relatively lower recognition of MD among medics in this study is somewhat unexpected. This warrants further investigation in future research to better understand the professional, cultural, or contextual factors that may influence how MD is perceived and reported within this discipline.

Consistent with previous research, we also observed variation in some clinical scenarios most frequently encountered by medics, nurses, and AHPs (Allen et al. 2013; Houston et al. 2013; Whitehead et al. 2015). These findings highlight the importance of recognizing that the sources of MD can differ significantly across professional groups. Such variation should be considered when designing workplace interventions aimed at identifying, preventing, or mitigating MD.

### Impact of MD on professional quality of life and professional retention

Consistent with previous research, we found that MD is associated with reduced professional quality of life and increased turnover intentions (Whitehead et al. 2015; Colville et al. 2019; Epstein et al. 2019). Higher levels of burnout and STS, both components of compassion fatigue, were consistently associated with higher intensities of MD. Notably, the intensity of MD showed a stronger association with STS than with burnout. This suggests that when professionals are unable to provide care that aligns with their ethical standards, the emotional toll of witnessing patient suffering may have a greater impact than the cumulative strain of these work-related demands.

Participants who identified with the experience of MD in this study were also more likely to have considered leaving or changing their position, highlighting MD as a potential threat to the sustainability of the MND workforce. While higher MD intensity was associated with turnover intentions in bivariate analysis, this relationship was no longer significant after accounting for burnout and STS in the multivariable model. This pattern suggests that the influence of MD on workforce attrition may be mediated through its contribution to compassion fatigue. However, this potential pathway warrants further investigation and validation in future research.

#### Addressing MD

Evidence for interventions addressing MD in healthcare remains limited, with existing studies often constrained by methodological weaknesses and variable designs (Morley et al. 2021; Amos and Epstein 2022). Nevertheless, findings from this and other research underscore the need for multilevel strategies that reflect the diverse causes and consequences of MD (Fantus et al. 2024).

At the system level, strategies should aim to reduce context-specific structural and procedural barriers, such as processes that delay timely referrals or impede care coordination. At the organizational level, efforts must prioritize cultivating open, supportive, and psychologically safe workplace cultures conditions shown to buffer the effects of ethical uncertainty and promote adaptive responses such as moral resilience and professional growth (Perni 2017; Morley et al. 2021). Interdisciplinary working environments that support open discussion of ethical challenges are likewise associated with lower levels of MD (Wocial et al. 2024). In the context of MND care, where clinical deterioration and death are inevitable despite best professional efforts, supportive workplace cultures are especially vital.

Educational interventions incorporating ethics training, communication skills development, and reflective practice represent some of the most common and promising approaches to addressing MD (Molazem et al. 2013; Monteverde 2016; Meziane et al. 2018; Abbasi et al. 2019) However, these programs vary widely in their theoretical underpinnings, content, and duration, which limits replication and comparability of outcomes (Morley et al. 2021). Targeted education focused on recognising and managing ethical tensions specific to MND care should however be considered. Finally, individual-level factors such as personal resilience and adaptive coping warrant further exploration for their potential to mitigate the emotional and psychological impact of MD and to enhance professional quality of life among those working in this demanding clinical context.

## Study strengths and limitations

The current study achieved a high completion rate, with over 80% of professionals who started the survey completing it in full. This may reflect the relevance of the survey topic and questions to this particular population, as well as the effectiveness of reaching the appropriate target group. Additionally, the time invested in pilot testing and incorporating feedback from professionals working in the field likely helped to refine the survey content and format, reducing the risk of survey fatigue and early drop-off.

There was the potential for selection bias as those who chose to participate may have had a particular interest in wellness or professional well-being, while those experiencing the highest levels of work-related stress may not have selected to participate in an additional work-related task. Additionally, the cross-sectional nature of the study limits causal inference and the ability to determine the directionality of observed relationships. Nonetheless, the findings provide a valuable foundation for future longitudinal research and contribute important initial data on the experience of healthcare professionals working in MND care.

## Conclusion

This study found evidence of MD affecting approximately two-thirds of healthcare professionals working in MND services across Europe who responded to the survey. The most frequently identified sources of distress were situated at the organizational and system levels, where structural barriers prevented clinicians from delivering care aligned with their professional and ethical standards. As a result, professionals often found themselves witnessing avoidable patient suffering and caregiver burden they felt powerless to alleviate. Higher intensities of MD were associated with lower professional quality of life, and those who identified with experiences of MD were more likely to have considered leaving or changing positions, either currently or in the past. These findings highlight the importance of recognizing MD as a significant risk to workforce well-being and retention in MND care. Future research should examine how professionals cope with and adapt to MD in this context and evaluate the effectiveness of both organizational- and individual-level interventions aimed at mitigating its impact.

## Supporting information

10.1017/S1478951526103058.sm001 Walls et al. supplementary material Walls et al. supplementary material

## Supplementary material

The supplementary material for this article can be found at https://doi.org/10.1017/S1478951526103058.

## Table 1 Long description

The table lists 24 statements describing situations that may cause moral distress for healthcare professionals caring for people with ALS/MND. Items cover professional challenges such as uncertainty about disease progression, pressure to provide unwanted or nonbeneficial interventions, limited training for future planning and end-of-life discussions, and difficulty offering emotional support or hope. Patient and condition factors include communication barriers affecting respect for wishes, cognitive or behavioural changes limiting beneficial care, unaddressed physical needs, poorly managed symptoms, late-stage presentation, and breakdowns in the patient–clinician relationship. Family-related statements focus on limited time or services to reduce caregiver burden, conflicting priorities between patient and family, and difficulty implementing care without caregiver support. Team factors include poor interprofessional communication, staffing vacancies, and dependence on other professionals or organisations to deliver interventions. System and organisational factors include restrictive policies, delays in aids or services, regional inequities in access, lack of equipment or bed capacity, limited appointment time forcing prioritisation, and unsafe caseload expectations. The table is a qualitative inventory of potential causes rather than a ranking or frequency report.

Navigate back to Table 1.

## Table 2 Long description

The table reports the number and percentage of 230 respondents by profession and by country. Allied healthcare professionals were the largest professional group at 114 people, about half of the sample. Within allied health, physiotherapists were most common at 39, followed by occupational therapists at 23 and speech and language therapists at 22; smaller groups included psychologists at 14 and several roles at fewer than 10. Nursing roles totaled 46, including 41 nurses and 5 nursing assistants, while medical professionals accounted for 70, about three in ten. By country, the United Kingdom had the most respondents at 63, followed by France at 54; Denmark had 24 and the Republic of Ireland had 22. Several countries contributed small numbers, including single respondents from Czechia, Norway, and Switzerland. Percentages are rounded, so totals may not add exactly to one hundred.

Navigate back to Table 2.

## Table 3 Long description

The table compares demographic and work-related characteristics across three professional groups (medical, nursing, and allied health professionals) and the overall sample. Median percentage of the working week spent with individuals living with MND differed strongly by group: overall 41 (IQR 60), medical 41 (54), nursing 70 (50), and AHPs 31 (40), with a very small p-value. Working pattern also varied: full time was most common overall (174 of 230), highest in medical (61 of 70) and lowest in AHPs (78 of 114), with a p-value below 0.05. Gender distribution differed markedly: males were 40 percent in medical (28 of 70) versus 15 percent in nursing (7 of 46) and 16 percent in AHPs (18 of 114), with a very small p-value; non-binary counts were very small across groups. Age category differed: no medical respondents were under 30, while AHPs had the largest under-30 share (17 of 114), and medical had more respondents aged 51 and over (27 of 70) than AHPs (20 of 114), with a very small p-value. Years of experience also differed: medical had fewer in 0 to 10 years (21 of 70) and more in 21 years or more (23 of 70) than nursing (6 of 46) and AHPs (9 of 114), with a very small p-value. Several other outcomes showed little or no evidence of group differences where tested: past consideration of leaving, absenteeism, and awareness or resonance with moral distress had p-values indicating no clear differences. Some sections use smaller denominators due to missing responses, so percentages for those rows reflect only respondents to that item.

Navigate back to Table 3.

## Table 4 Long description

The table compares participant counts and median responses across three groups based on whether they resonated with a moral distress description: yes, no, or not sure. Most participants were in the yes group (128, 67%), with 46 (20.5%) in no and 28 (12.5%) in not sure. The median number of statements endorsed was higher for the yes group (16) than for both the no and not sure groups (10 each). Median moral distress thermometer scores were also higher for the yes group at 4, labeled uncomfortable, compared with 2, labeled mild, for both no and not sure. Group differences for both the number of statements endorsed and thermometer scores were reported as statistically significant with a p-value less than 0.001. Medians summarize typical responses and do not show variability within each group.

Navigate back to Table 4.

## Table 5 Long description

Endorsement rates are reported for 24 statements about challenges in ALS or MND care, shown as number and percent saying yes overall and within medical, nursing, and allied health professionals, with a rank for each group and a p-value for between-group differences. The most widely endorsed issue was seeing people struggle while waiting for necessary aids, devices, or services, reported by 196 of 220 respondents (89%) and ranked first in every professional group. Other highly endorsed items overall included unequal access by region (77%), unaddressed physical care needs due to psychological adjustment difficulties (71%), distress or pain from poorly managed symptoms (71%), and inability to reduce caregiver burden due to limited time or services (70%). Several statements differed by professional group: allied health professionals more often reported uncertainty about disease trajectory affecting future planning (61% AHPs versus 42% medical and 40% nursing) and lack of training for future planning conversations (40% AHPs versus 20% medical). Medical respondents most often reported witnessing distress or pain from poorly managed symptoms (84% medical versus 54% nursing and 67% AHPs). Nursing respondents more often reported difficulty implementing care without family or informal caregiver support (69% nursing versus 40% medical and 53% AHPs). Differences should be interpreted cautiously because the p-values reflect group comparisons and do not indicate the size or practical importance of the differences.

Navigate back to Table 5.

## Table 6 Long description

The table lists the five highest rated workplace stressors reported by three professional groups supporting people with ALS or MND: medical staff, nursing staff, and allied health professionals. All three groups include delays in obtaining necessary aids, devices, or services, unequal access to services by region, and feeling unable to reduce the burden on family or informal caregivers due to limited time or services. Medical staff uniquely highlight distress or pain from poorly managed symptoms, while nursing staff uniquely emphasize being unable to provide needed emotional support or comfort to patients and families. Nursing and medical groups both cite compromised care caused by shortages in equipment, resources, or bed capacity, whereas AHPs instead include concerns about respecting patient wishes when communication problems occur, difficulty communicating hope without a cure, and inability to provide beneficial interventions due to cognitive or behavioural changes. Overall, the shared pattern points to system capacity and access issues as common drivers of stress, with group-specific stressors reflecting different clinical roles and responsibilities.

Navigate back to Table 6.

## Table 7 Long description

The table reports associations between staff characteristics and the Moral Distress Thermometer total score using both bivariate tests and an adjusted linear regression. In bivariate analyses, moral distress increased with a higher percentage of the work week spent with ALS or MND, higher burnout, and higher secondary traumatic stress; compassion satisfaction was associated with lower moral distress. Gender showed a small bivariate difference, while profession group and years of experience did not show clear bivariate differences. In the adjusted model, burnout remained positively associated with moral distress, and secondary traumatic stress showed a large positive association with moral distress. Adjusted differences by profession group, gender, and experience were small and had wide confidence intervals that included no effect. Inpatient setting, turnover intention, and absenteeism had positive or negative adjusted estimates, but their confidence intervals included no effect. Some variables were only assessed bivariately or were not carried into the adjusted model, so not all bivariate findings can be compared directly with adjusted results.

Navigate back to Table 7.

## Figure 1 Long description

The image A showing a bar graph titled Have you Heard of the Concept Moral Distress Before question mark. Legend: Yes left parenthesis percent right parenthesis, No left parenthesis percent right parenthesis. Y axis label Percentage, range 0 to 60. X axis categories: Overall, Medical, Nursing, A H P. Values shown: Overall Yes 47, Overall No 53. Medical Yes 50, Medical No 50. Nursing Yes 52, Nursing No 48. A H P Yes 43, A H P No 57. The image B showing a bar graph titled Does this Description of Moral Distress Resonate with Your Experience of Working with M N D question mark. Legend: Yes left parenthesis percent right parenthesis, No left parenthesis percent right parenthesis, Not Sure left parenthesis percent right parenthesis. Y axis label Percentage, range 0 to 80. X axis categories: Overall, Medical, Nursing, A H P. Values shown: Overall Yes 67, Overall No 21, Overall Not Sure 13. Medical Yes 56, Medical No 27, Medical Not Sure 17. Nursing Yes 70, Nursing No 23, Nursing Not Sure 7. A H P Yes 75, A H P No 14, A H P Not Sure 11.

Navigate back to Figure 1.
