# Supplementary material for: Moral distress in healthcare professionals working with motor neuron disease
Source: Palliat Support Care. 2026 Jul 15;24:e196. doi: 10.1017/S1478951526103058 (PMC13430491; doi:10.1017/S1478951526103058)
Supplement: Walls et al. supplementary material [file S1478951526103058sup001.docx]

**Supplementary Material:**

**Supplementary Material 1:** Professional Quality of Life Scale Scores and Classification

## Professional Quality of Life Outcomes:

| ProQOL-5 Sub-Scales  (*n* = 217) | Low  (≤ 22) | Moderate  (23 – 41) | High  (≥ 42) |
| --- | --- | --- | --- |
|  | n (%) | n (%) | n (%) |
| Burnout | 113 (52) | 104 (48) | 0 (0) |
| Secondary Traumatic Stress | 141 (65) | 75 (34.5) | 1 (0.5) |
| Compassion Satisfaction | 0 (0) | 143(66) | 74 (34) |

Subscale scores from the Professional Quality of Life Scale (ProQOL-5) classified according to established cut-off ranges, with respondents grouped into low, moderate, or high categories based on their scores (Stamm, 2010).

**Supplementary Material 2:** Correlation Matrix

|  | Compassion Satisfaction  (ProQOL) | Secondary Traumatic Stress (ProQOL) | Burnout (ProQOL) |
| --- | --- | --- | --- |
| MD Thermometer | - 0.25*** | 0.53*** | 0.47*** |
| Burnout | -0.57*** | 0.65*** | - |
| Secondary Traumatic Stress | -0.27*** | - | - |

Pairwise spearman rank-order correlation coefficients between MD Thermometer and Pro-QOL outcomes**<0.05, **<0.01, ***<0.001*

**Supplementary Material 3:** Frequency and Rank Order of Statements Identified as Most Distressing

| Rank | Frequency(n) | Statement | Statement |
| --- | --- | --- | --- |
| 1 | 82 | 20 | Witness individuals with ALS/MND struggling while waiting for necessary aids, devices, or services. |
| 2 | 72 | 21 | Experience individuals with ALS/MND receiving unequal access to services/resources depending on the region where they live. |
| 3 | 64 | 12 | Feel unable to alleviate the burden on family and informal caregivers because of insufficient time or available services. |
| 4 | 62 | 22 | Witness compromised care for individuals with ALS/MND due to lack of equipment/resources/bed capacity. |
| 5 | 59 | 6 | Have difficulty communicating hope or optimism for individuals with ALS/MND with no available cure for the disease. |
| 6 | 52 | 10 | Witness individuals with ALS/MND in distress or pain due to poorly managed symptoms. |
| 7.5 | 50 | 5 | Feel unable to offer individuals with ALS/MND and their families the emotional support or comfort they need. |
| 7.5 | 50 | 8 | Feel unable to provide an intervention that could benefit the individual with ALS/MND because of cognitive or behavioural changes. |
| 9.5 | 49 | 7 | Worry that the wishes of individuals with ALS/MND are not being respected when problems with their communication arise. |
| 9.5 | 49 | 11 | Struggle to provide the necessary care for someone whose condition is already at an advanced stage when I first meet them. |
| 11 | 47 | 9 | Witness physical care needs that remain unaddressed because of difficulties with psychological adjustment. |
| 12 | 46 | 17 | Witness care for individuals with ALS/MND being negatively affected because of staffing vacancies within a team or service. |
| 13 | 39 | 18 | Have difficulty providing an intervention due to reliance on another healthcare professional/organisation also involved in the person’s care. |
| 14.5 | 36 | 4 | Feel that by initiating future care planning, I am adding to the individual’s psychological distress. |
| 14.5 | 36 | 16 | Witness compromised care for individuals with ALS/MND because of poor communication between healthcare professionals. |
| 16 | 34 | 14 | Struggle to meet the needs of both individuals with ALS/MND and their family members because of their conflicting priorities or opinions. |
| 17 | 32 | 2 | Feel pressure to provide care/intervention that I don't believe will benefit the individual with ALS/MND at the request of others, such as family, senior colleagues, or other services. |
| 18 | 29 | 19 | Struggle to meet the needs/expectations of individuals with ALS/MND or their families due to limitations imposed by my organisation's policies or procedures. |
| 19 | 27 | 1 | Feel unable to effectively plan for the future care of individuals with ALS/MND because of my uncertainty regarding the trajectory of disease progression. |
| 20 | 25 | 3 | Feel unprepared to engage in future care planning or end-of-life care conversations due to a lack of appropriate training. |
| 21 | 22 | 24 | Be required to care for more individuals with ALS/MND than I can safely care for. |
| 22 | 20 | 23 | Feel restricted to prioritise only certain aspects of care for individuals with ALS/MND due to limited time during appointments. |
| 23 | 17 | 15 | Have difficulty implementing care or interventions without family or informal caregiver support. |
| 24 | 7 | 13 | Feel unable to effectively deliver care for an individual with ALS/MND due to a breakdown in the patient-clinician relationship. |
